# Data spaces and self-sovereign identity: Ecosystem landscape and a systematic literature review on Gaia-X-aligned data spaces

**DOI:** 10.1016/j.dib.2026.112668

**Published:** 2026-03-12

**Authors:** Roi Sánchez Serna, Carla López García, Ana I. González-Tablas

**Affiliations:** Universidad Carlos III de Madrid, Dpto. de Informática, COSEC Lab, Av. de la Universidad, 30, Leganés, 28911, Madrid, Spain

**Keywords:** Data spaces, Gaia-X, Identity management, Credential management, Digital sovereignty, Interoperability, Standards, Systematic literature review

## Abstract

Data spaces have emerged as a cornerstone of the European data strategy, aiming to enable secure, sovereign, and interoperable data sharing across sectors and borders. Within this vision, identity and credential management are fundamental to ensuring trust, compliance, and usability. Within the European context, Gaia-X is emerging as the fundamental trust framework for data spaces. However, the complexity of the data spaces ecosystems poses significant barriers to understanding the concepts and technologies behind the Gaia-X framework, which in turn hinders its adoption. In this paper we first elaborate an ecosystem landscape overview, identifying the current state of the key aspects of identity and credential management in dataspaces, with a particular attention to Gaia-X’s contributions and role; and then we conduct a systematic literature review of recent academic work addressing identity and credentials in data spaces, paying again special attention to those aligned with the Gaia-X framework. Our analysis shows that most contributions remain at a high conceptual and architectural level, while standards remain fragmented and under continuous development, leading to ongoing interoperability challenges. We conclude that significant progress is still required in the previous areas, but also identify a set of new research direction.

## 1 Introduction

Data spaces are a key EU policy strategy, backed by substantial funding and seen as a critical instrument for advancing the European data economy. Data spaces inspired in European values are based on the foundational pillars of trust, compliance, interoperability, and sovereignty, and are actively encouraged to foster participation in the data ecosystem among both industry and users. However, the ecosystem’s inherent complexity means that standards, guidelines, and documentation are still evolving, creating barriers to entry and friction in collaboration due to novelty, fragmentation, and distributed documentation.

In this context, Gaia-X [1] is emerging as a fundamental, decentralized digital trust framework for data spaces that are aligned with European values. It offers a concrete focus on identity and trust management through its architecture and governance mechanisms. A critical early step in deploying a Gaia-X compliant data space is understanding identity and credential management, and trust governance under Gaia-X rules. Although Gaia-X provides documentation, guides, and proof-of-concept software and other resources, achieving a comprehensive yet practical understanding of how to integrate Gaia-X into the design and deployment of a data space remains challenging. Organizations pursuing this goal must consider not only the functions and roles that Gaia-X provides within the current data space reference architectures, but also how these functions are implemented in practice. Failure to understand this may lead to increased costs, delayed delivery, and eroded stakeholder confidence; to tackle this, a natural first step is to analyse how other data spaces address these issues.

Therefore, this work has the goal of advancing in the understanding the previous questions. The approach taken is two fold: (a) to elaborate an ecosystem landscape overview, identifying the current state of the key aspects of identity and credential management in dataspaces, with a particular attention to Gaia-X’s contributions and role; and (b) to conduct a systematic literature review of recent academic work addressing identity and credentials in data spaces, paying again special attention to those aligned with the Gaia-X framework.

*Related Work* Systematic reviews have examined how identity and credential mechanisms are integrated into data spaces with references to Gaia-X. Schmidt et al. [2] present a review complemented by expert interviews that identifies major challenges for sovereign data exchange and maps privacy-enhancing and authenticity-enhancing technologies to these challenges. They emphasize that access and usage control are central issues and argue that combinations of technologies such as verifiable credentials, zero-knowledge proofs, usage control policies, multiparty computation, and trusted execution environments are required to support identity assurance, accountability, and regulatory compliance in federated data spaces. Wicaksono et al. [3] focus on digital product passports as a key application of data spaces in the circular economy. Their review identifies relevant architectural layers, connectors, and semantic technologies, and stresses the importance of Gaia-X standards and connectors for interoperability. Identity and credential management are addressed through decentralized identifiers, verifiable credentials, and distributed ledger infrastructures that enable provenance and attestation. However, although these works consider Gaia-X, they do not analyze authentication aspects in detail, which is the specific focus of our contribution.

Other surveys have taken a broader perspective on digital identity and self-sovereign identity (SSI). Schumm et al. [4] provide a comprehensive study of decentralized identity applications, going beyond academic literature to include grey literature and real-world deployments. Their work highlights barriers to adoption of decentralized identities, such as lack of standardization, governance issues, and interoperability gaps. Krul et al. [5] conduct a systematization of knowledge (SoK) on trust in SSI, proposing three trust models that capture the threats and mitigations identified across literature and implementations. Their framework catalogs SSI components, identifies shortcomings in existing systems, and provides design requirements for trust. Similarly, Bacco et al. [6] present a systematic survey of data spaces as a whole, outlining their architectural dimensions, governance models, and future research directions.

In summary, prior work has either focused on general architectures of data spaces [6], on decentralized identity adoption challenges [4], or on conceptual trust models in SSI [5]. Our work, by contrast is distinct in that it specifically addresses identity and credential management with a particular focus on the aspects affected by the Gaia-X framework, examining how these data spaces integrate self-sovereign identity mechanisms and how they align with regulatory and interoperability requirements.

*Contributions* Our main contributions are:

• A comprehensive overview of the ecosystem landscape, organized in three parts: data spaces; self-sovereign identity (SSI); and Gaia-X. The Gaia-X section not only describes Gaia-X’s Trust Framework but also maps its components and technologies to the data space and SSI ecosystems.
• A synthesis of the academic literature on the practical design and implementation of Gaia-X's aligned data spaces, achieved through a Systematic Literature Review (SLR) focused on identity and credential mechanisms in data spaces aligned with Gaia-X. The findings provide a critical synthesis, revealing that most contributions remain at a high conceptual and architectural level, and that standards are fragmented and still evolving, resulting in ongoing interoperability challenges. Building on the SLR and the ecosystem overview, we identity a set of promising research directions.

*Structure of the article* In terms of structure, this paper is organized into several key sections. Section 2 introduces the concept of data spaces and their current development; Section 3, which explores the broader framework of digital identity management and SSI. Section 4 examines Gaia-X and its relationship with data spaces and SSI. Section 5 presents the Systematic Literature Review, offering a synthesis of existing research, discussing the main findings and offering a list of promising future research directions. Finally, Section 6 discusses the main findings and outlines, reflecting on the implications for future research and practice.

## 2 Data Spaces: Definition and Current State

This section outlines the concept of data spaces, their evolution, and the main components that characterize them. It situates Data Spaces within the European strategy for data, highlighting key regulations (GDPR, Data Governance Act, Data Act), as well as EU initiatives such as DSSC or SIMPL. Finally, it introduces the organizations and standardization —including IDSA, FIWARE, Gaia-X, BDVA, and others— that contribute to shaping their current development.

### 2.1 What is a data space?

A data space is an interoperable framework, based on common governance principles, standards, practices and enabling services, that enables trusted data transactions between participants [7]. It is built upon common governance principles, standards, and enabling services that ensure compliance with regulations and fair treatment of all parties. Data spaces support decentralized collaboration by defining roles, responsibilities, and procedures through a governance framework, thereby maintaining data sovereignty and trust. They facilitate structured data interactions through technical, legal, and organizational mechanisms, enabling value creation across different contexts.

The philosophy behind data spaces is to enable sovereign, secure, and interoperable data sharing across organizations without requiring central control or full data integration. They promote a paradigm of data collaboration where participants retain control over their assets while benefiting from discoverability, trust, and value creation within federated ecosystems.

Data spaces face multiple challenges such as data interoperability and data value [8]. Interoperability remains difficult because heterogeneous and siloed sources must be aligned across ontologies, schemas, and entities, while legacy systems raise the integration cost and data sovereignty often forces a staged harmonization with semantic alignment preceding instance-level integration. Data value is also nontrivial, since even with unified access, extracting insights requires data cleaning, feature engineering, and advanced analytics; although semantic annotations and automated machine learning can reduce the effort, practical obstacles persist, including dynamic data availability, transfer of knowledge across heterogeneous contexts, and explicit handling of uncertainty in model outputs.

### 2.2 Brief history of data spaces

The concept of data space was first introduced around 2005 as a response to the increasing heterogeneity and volume of data in digital systems [6]. Unlike traditional database systems, which rely on strict schemas and centralized control, dataspaces were envisioned as flexible ecosystems for data sharing and management across distributed sources, without enforcing tight integration. Early work emphasized the distinction between data spaces and databases, highlighting how the former could accommodate loosely coupled, heterogeneous datasets using so-called Dataspace Support Platforms (DSSPs) [9]. This approach shifted the paradigm from schema-first to data-first, acknowledging the limitations of conventional Data Base Management Systems (DBMSs) when dealing with dynamic and domain-agnostic data environments.

Over the following two decades, the concept evolved significantly. From the original exploratory studies and prototype systems, data spaces have entered a phase of consolidation, driven by standardization efforts and practical deployments. Spatial Data Infrastructures (SDIs) [10] and initiatives such as INSPIRE^1^ in the EU paved the way by demonstrating the value of federated and interoperable data systems. Recently, the emergence of architectures and frameworks—like the International Data Spaces (IDS) Reference Architecture Model [11] and the Data Spaces Support Centre^2^ (DSSC) Blueprint [12]— has accelerated the transition from conceptual models to operational systems. These developments aim to ensure data sovereignty, support FAIR (Findability, Accessibility, Interoperability, and Reuse) principles, and enable flexible, trustworthy data exchange across sectors and borders.

In recent years, in Europe data spaces have gained strong momentum due to growing political and economic interest in trusted data exchange and digital sovereignty. There are multiple large-scale European initiatives –both industry and institution led– that have mobilized resources and defined common strategies to accelerate adoption. Notable examples include the GAIA-X AISBL^3^ launched in 2019, the Data Space Business Alliance^4^ (DSBA) in 2021, and the Data Spaces Support Centre (DSSC) in 2022. These efforts are also supported by the development of open-source software (e.g., the Eclipse Dataspace Connector or the main elements of the Gaia-X Digital Clearing House). As a result, data spaces are moving from exploratory pilots to scalable infrastructures intended to underpin data-driven innovation and cross-sector collaboration.

### 2.3 Main components of data spaces

**Fig. 1:**
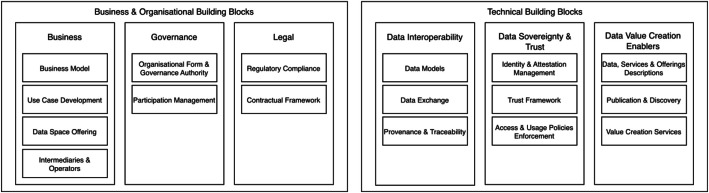
Overview of data spaces building blocks. Adapted from [12].

The main components of a data space are designed to support secure, sovereign, and interoperable data exchanges between participants. Currently, data spaces are seen as highly complex systems that need to integrate and coordinate several subsystems. The DSSC provides in its Data Spaces Blueprint v2.0 [12] an overview of the business, organisational and technical building blocks of a data space, illustrating the complexity (see Fig. 1).

**Fig. 2:**
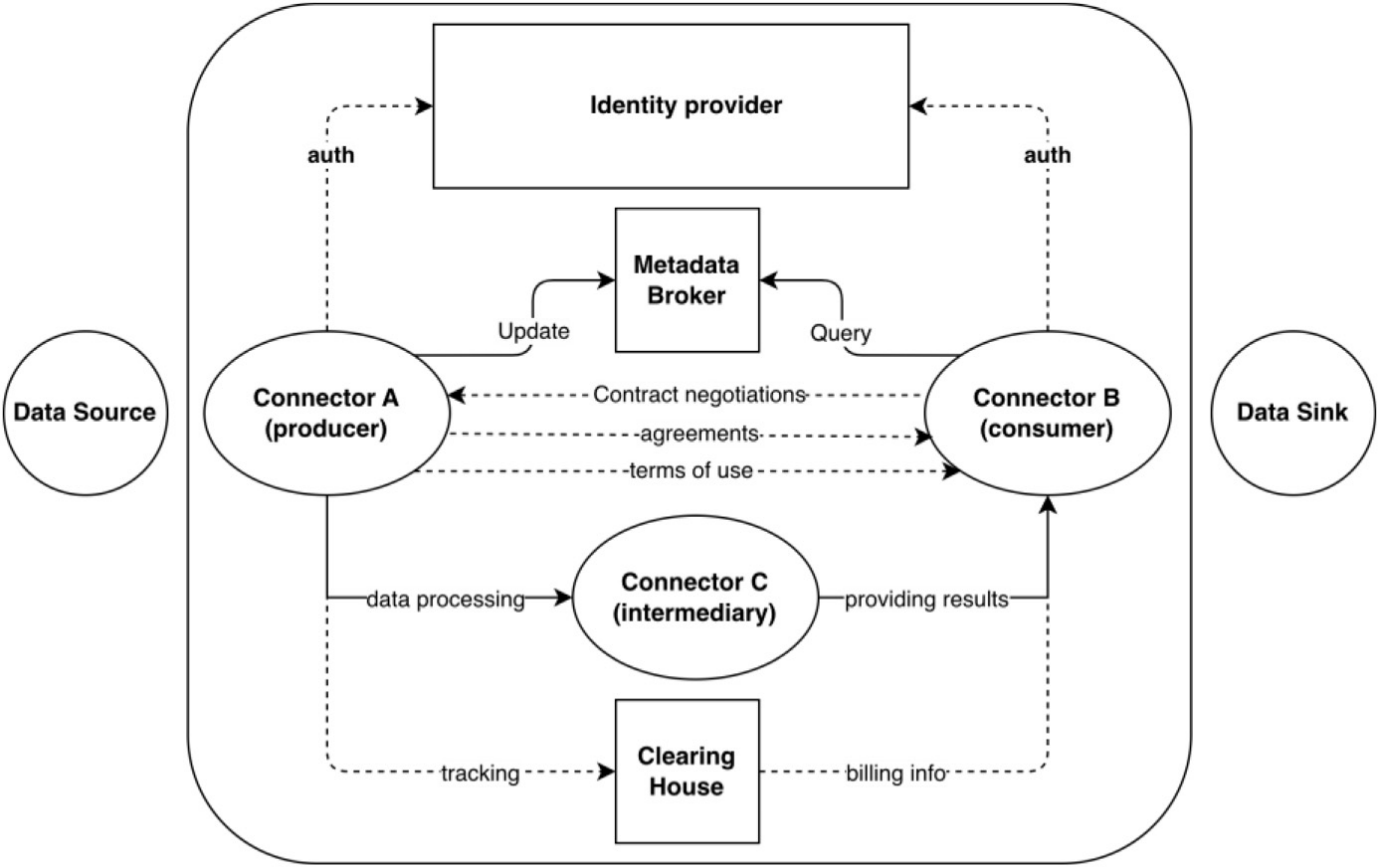
Interactions among the main technical components in a data space. Adapted from [6].

Although existing data space reference architectures may define their components slightly differently, most converge on the main technical elements involved in the data exchange. In their recent work [6], Bacco et al. review what a data space is and summarize its main technical components and interaction flows (see Fig. 2). At the core of a data space lies the connector, a software component deployed at each participant’s endpoint that manages the communication with data sources, metadata, and usage policies. Connectors follow a control/data plane separation model, handling identity verification, and metadata discovery is enabled through a metadata broker, which supports publish-subscribe mechanisms for asset discovery. The catalogue component allows providers to expose data assets and their associated policies, while a clearing house logs transactions and manages billing information. Additional components include policy enforcement services, vocabulary providers for semantic interoperability, and application stores for distributing executable logic within the ecosystem.

### 2.4 European strategy for data spaces

***Policies and regulations*** The European Strategy for Data Spaces is a core component of the broader European Strategy for Data, conceived within the EU’s data economy agenda to unlock secure, cross-border data sharing while preserving sovereignty. Building on this vision, the Data Spaces initiative focuses on establishing common, sector-specific data spaces (e.g., health, energy, manufacturing) under interoperable and trustworthy governance. Key elements of the overarching strategy include a unified data market, data governance and sovereignty, and wide data sharing; the data spaces add the practical layer of sectoral, interoperable infrastructures designed to enable secure data access, reuse, and collaboration across borders. Next, we briefly describe the main facts about the European Strategy for Data, the Data Governance Act and the Data Act, which provide the regulatory framework for European Data Spaces.

*European Strategy for Data* (2020) [13] The European Strategy for Data, presented in 2020, defines the road-map of the EU to establish a single market for data and to strengthen digital sovereignty. Its objective is to maximize the value of data by ensuring its availability, accessibility, and reuse across all sectors. The strategy also covers regulatory principles (such as the Data Governance Act and the Data Act), the deployment of open standards, and mechanisms for interoperability. The strategy is structured around four key pillars:

• First, a cross-cutting governance framework regulates access to and use of data, promoting consistency among Member States and fostering tools such as regulatory sandboxes.
• Second, it promotes technological enablers and investments in federated cloud and edge computing infrastructures, interoperable data-sharing tools, and open standards.
• Third, the strategy emphasizes the development of skills and capacities, including data literacy, the strengthening of portability rights, and the deployment of personal data spaces.
• Finally, it promotes the creation of common data spaces in strategic sectors like health, public administration, energy, or finance. These data spaces combine governance frameworks and interoperable infrastructures to enable cross-border and cross-sectoral data use.

With this, the European Strategy for Data lays the foundation for a competitive and innovative ecosystem, reinforcing Europe’s leadership in the data economy. Although the strategy is supported by the usual EU’s funding mechanisms, its core funding comes from the Digital Europe Programme (DEP).

*Data Governance Act* (2022) [14] The Data Governance Act (DGA), adopted by the European Union in 2022 and in force since September 2023, represents a fundamental component of the European Data Strategy. Its objective is to increase the availability and responsible use of data in Europe by establishing mechanisms of trust among stakeholders and ensuring that data sharing respects the European values of sovereignty, transparency, and rights protection.

The regulation creates a framework for the re-use of public sector data, particularly when subject to third-party rights such as confidentiality, privacy, or intellectual property. It also introduces the role of data intermediaries, neutral entities that facilitate the secure and transparent exchange of information between organizations and individuals, and promotes data altruism, enabling citizens and companies to voluntarily share their data for purposes of general interest such as scientific research, innovation, or public policy.

In addition, the DGA establishes the European Data Innovation Board (EDIB) to coordinate the implementation of the regulation and foster interoperability across different data spaces. By doing so, it provides a governance framework that enhances transparency, trust, and control, positioning itself as a key element in the development of a strong and sustainable European data ecosystem.

*Data Act* (2023) [15] The Data Act, adopted in 2023, establishes a regulatory framework governing the access, use, and sharing of data particularly generated by the Internet-of-Things services. Its objective is to ensure that users of digital devices and services have the right to access the data they generate and that such data can be shared, under clear conditions, with third parties.

The regulation specifically addresses B2B (business-to-business) and B2G (business-to-government) data-sharing models. In the B2B context, it requires that data-sharing agreements be transparent and non-discriminatory, introducing safeguards to prevent contractual abuses. In the B2G context, it stipulates that certain public authorities may access private data in situations of public interest, such as emergencies or disasters.

The Data Act also introduces measures to strengthen interoperability and portability in cloud services, imposing the obligation to allow switching between providers and limiting vendor lock-in practices. Overall, the regulation establishes a technical and legal framework that promotes access, reuse, and circulation of data across the EU, representing a key instrument for the deployment of interoperable and sovereign data spaces.

*Other relevant European regulations* Although tangential to this work, other relevant European Union regulations affecting data-space development must be considered when designing and deploying a data space, such as the General Data Protection Regulation (GDPR) [16], the NIS2 Directive [17], the Cybersecurity Act [18], or the Interoperable Europe Act [19].

***EU Supported Initiatives*** The EU is funding several sector and domain-specific initiatives focused or related to data spaces. Next, we review the most relevant for the objectives of this work.

*Common European Data Space*s^5^ Common European Data Spaces aim to facilitate access to and reuse of data within secure and trustworthy environments, enhancing innovation across strategic sectors in the EU. Initiated by the European strategy for data in 2020, these spaces are being developed in areas such as health, energy, agriculture, finance, manufacturing, and cultural heritage, among others. They are built upon shared infrastructures and governance mechanisms that enable data pooling and exchange while respecting EU rules on privacy, consumer protection, and competition.

The initiative is grounded in the idea that stakeholders shape each data space according to sector-specific needs, while common frameworks ensure interoperability and fair access. Data holders retain control over their data and can choose whether to share it freely or for compensation. These spaces are expected to foster the creation of new data-driven services and products, reinforcing the digital single market. Development is ongoing across fourteen sectors, with support from EU programmes such as Digital Europe and Horizon Europe Programmes. Among the most developed examples are the Health Data Space, which facilitates cross-border sharing of medical records and genomics data, and the Mobility Data Space, which supports the interoperability of transport services through harmonized data platforms. Both examples demonstrate how sector-specific ecosystems can be created under common principles to foster data-driven services and applications.

*Data Spaces Support Center (DSSC)* The Data Spaces Support Centre (DSSC) is an initiative of the European Commission, funded under the Digital Europe Programme (DEP), and which acts as a reference, providing methodologies, guidelines, and tools that help organizations design and deploy their own data spaces in line with European principles of sovereignty, transparency, and trust. It is aimed at both the public sector and companies wishing to develop sovereign and interoperable data spaces. Moreover, the DSSC collects and recommends existing standards, specifications, and best practices to enable different data spaces to interoperate with each other. It also promotes governance models and frameworks that ensure regulatory compliance.

Among its main resources is the Blueprint [12], which serves as a guide for organizations wishing to create or participate in data spaces, recommending standards and best practices. The Blueprint proposes starting with the key concepts and definitions of data spaces, and then introduces the building blocks, which are intended to break down data spaces into smaller and more manageable components. These can be implemented and combined to achieve the full functionality of a data space. The building blocks are divided into two broad groups (see Fig. 2): business and organizational building blocks (including business, governance, and legal) and technical building blocks (covering data interoperability, data sovereignty & trust, and data value creation enablers).

In addition, the DSSC provides the Toolbox, a catalogue of tools for data spaces that includes connectors, digital wallets, vocabulary hubs, contract templates, and many other components required to set up, participate in, and operate a data space. These tools are organized into categories aligned with different stages: preparation, validation and submission, publication, and monitoring.

*SIMPL: Smart Open-Source Middleware*^6^ SIMPL is a European Commission procurement initiative designed to provide common, modular, and open digital infrastructure for data spaces, and particularly for EU-funded ones. . It acts as a middleware platform to enable interoperability, security, and trust in cross-border data exchange. Based on open-source principles, SIMPL offers reusable and standardized components that reduce technological fragmentation and promote the adoption of common data sharing practices, fully aligned with the objectives of the European Data Strategy. While the DSSC’s role is to guide in the design and deployment of EU-funded data spaces, SIMPL can be viewed as the provider of a common technical infrastructure of such spaces.

The initiative offers three main products: SIMPL-Open, the open and modular core, enabling the deployment of reusable components and the adoption of common standards; SIMPL-Labs, a testbed environment for experimenting with requirements and prototyping before production development; and SIMPL-Live, a real-word implementation of the middleware applied to specific data spaces. In this initial phase, SIMPL has launched a Minimum Viable Product (MVP) with some essential functionalities such as participant onboarding, service publication, and basic interoperability management. This progressive approach enables validation of the architecture and positions SIMPL as a reference for building an interoperable and trustworthy European data ecosystem.

*DIGIT B, SEMIC and eGovERA* The European Commission fosters digital interoperability through several interrelated initiatives, such as eGovERA,^7^ SEMIC,^8^ and DIGITB.^9^ These initiatives are complementary and together form a coherent ecosystem that supports the development of interoperable digital public services across the EU. SEMIC was an initiative to promote semantic interoperability among public administrations. It provides a platform to share and reuse existing specifications, reducing development costs and increasing system interoperability. DIGIT B is an initiative of SEMIC that provides open data models, semantic specifications, and tools to ensure interoperability within and between data spaces. It enables the definition of building blocks and data exchange protocols. Additionally, it offers support for data validation and conformance, ensuring that information is understandable and usable across different domains and applications. Lastly, eGovERA is an initiative that provides public administrations with detailed reference models and tools for designing interoperable Digital Public Services. Based on its European Interoperability Reference Architecture (EIRA), eGovERA offers a structured approach for creating integrated, EU-compliant solutions in various fields.

### 2.5 Organizations and standards

***Organizations*** In addition to the regulation that has been developed and the initiatives directly funded by the European Commission, a number of organizations—some operating under the EU umbrella—are strongly pushing the development of data spaces and their adoption by industry and public administrations in Europe. A list of the most representative organizations for the purpose of this paper is presented below.

*International Data Spaces Association (IDSA)*^10^ The International Data Spaces Association (IDSA) participates as a key partner in numerous initiatives that implement and extend data space principles. Its contributions span reference architectures [11], governance models, legal and ethical compliance, and technical interoperability. These efforts support the creation of scalable, secure, and standardized data ecosystems. For instance, in the Health-X Dataloft^11^ project, IDSA contributes to building an infrastructure for the secure sharing of health data with patient-centric control. In the Omega-X^12^ project, IDSA helps develop a federated energy data space that supports multi-vector energy flows and ensures transparency and sovereignty through open standards. These projects reflect IDSA’s role in translating conceptual models into practical, cross-sector data solutions.

*FIWARE Foundation*^13^ The FIWARE Foundation is a non-profit organization established in 2016 to promote the use of open standards and technologies which facilitate the development of smart solutions. Its technological approach is based on a set of open-source components known as Generic Enablers (GEs), which act as connectors within platforms, enabling the management, exchange, and secure handling of data in an interoperable ecosystem.

Among these components, two are particularly relevant. The Context Broker (e.g., Orion,^14^ considered the core of FIWARE, manages context information in real time and allows applications and services to create, query, and update data, among other operations. The Connector, in turn, focuses on interoperability and data sovereignty in data exchange, ensuring that communication between different organizations or data spaces takes place securely. FIWARE also defines open APIs, in particular NGSI and its evolution NGSI-LD,^15^,^16^ under the ETSI ISG CIM initiative. These interfaces enable the integration of data from multiple sources, the addition of semantics, and the creation of knowledge graphs. Within the framework of European data spaces, FIWARE thus provides the technological layer that makes possible the reliable exchange of information among multiple participants.

*GAIA-X European Association for Data and Cloud AISBL* The Gaia-X European Association defines a common framework for a European data and cloud infrastructure built on the principles of digital sovereignty, transparency, interoperability, and trust. It does not directly provide technological infrastructure but establishes the Gaia-X Trust Framework,^17^ which sets conformity criteria and certification mechanisms to ensure that services and providers are recognized as Gaia-X compliant.

A central element of this approach is the Gaia-X Digital Clearing House,^18^ which functions as a certifying and trust entity. Its role is to independently verify that providers and services meet the specifications, thereby ensuring transparency and reliability within the ecosystem. Another key mechanism is the Gaia-X Labels,^19^ a labeling system that classifies providers according to their level of compliance with European principles of sovereignty, portability, and transparency. These labels enable organizations and users to easily identify which services meet the trust requirements established by Gaia-X.

*Big Data Value Association*^20^ *(BDVA)* The Big Data Value Association (BDVA) is an international non-profit organization headquartered in Brussels, founded in 2014 as the private counterpart of the European Commission in the Big Data Value Public-Private Partnership (BDV PPP). Its mission is to foster innovation in data and artificial intelligence (AI) and to build a sustainable, competitive European ecosystem aligned with European values.

One of the key initiatives of the BDVA is the i-Spaces Federation,^21^ which certifies European data and AI innovation hubs through the i-Space label, a quality seal that guarantees compliance with strict criteria regarding infrastructure, services, business strategy, impact, ethics, and federation. BDVA also operates through specialized Task Forces that bring together members to address topics such as standardization, data governance, or trustworthy AI. Particularly, BDVA has a specific Task Force focused on data spaces (Data Spaces Task Force). Furthermore, the BDVA identifies the main legal, technical, and interoperability challenges and proposes strategic objectives aligned with the European Commission. Through projects such as DataPorts^22^ and i3-Market,^23^ it contributes to the development of a common reference architecture for data spaces. Overall, the BDVA acts as a strategic driver connecting research, industry, and policy to foster a trustworthy and democratic European data economy.

*Eclipse Foundation*^24^ The Eclipse Foundation is an international non-profit open-source software organization which develops practical tools in the context of data spaces. One of its main initiatives is the Eclipse Dataspace Components^25^ (EDC), a reference framework providing architecture, code, and components, which includes core elements such as connectors, federated catalogs, or identity services, and has been adopted in large-scale projects such as Catena-X and Eona-X.

In addition, it drives the Eclipse Dataspace Working Group, which promotes the global adoption of these technologies and their alignment with standards such as those of Gaia-X and IDSA. Within this framework, key extensions have been developed, including the Dataspace Protocol [20], Decentralized Claims Protocol (DCP) [21] and the Data Rights Policy Profile^26^ (DRP), which is being developed and establishes rules during data transactions. Finally, the Eclipse Technology Compatibility Kit (TCK) guarantees the conformity of implementations with specifications, providing an independent validation mechanism.

*TM Forum*^27^ TM Forum is a global association which defines standards and best practices that enable interoperability and digital transformation. It features the Data Collaboration Reference Architecture (GB 1050, v3.0.0), which outlines how to share and reuse through catalogs, roles, and standardized APIs. It also provides the Information Framework (SID), a reference model and common vocabulary that defines key entities and a common data model dictionary. Additionally, the Open Digital Architecture (ODA), which offers modular components and open APIs to modernize systems, facilitating the integration of digital services.

***Standards*** Finally, standardization bodies are currently developing specifications related to data spaces. Next, we list those that are expected to influence the EU landscape.

*CEN/CENELEC* One of the missions of the CEN/CENELEC committee is to develop and define standards that promote interoperability, security, and trust across different sectors like data spaces. Recently CEN has published two key documents for data spaces, that define the requirements for trustworthy and secure data transactions. CWA 18125:2024 [22] defines core concepts, terminology, and mechanisms for trustworthy data exchange, regardless of architecture or technical implementation. CWA 18245:2025 [23] complements the first part by defining the trustworthiness requirements that data transactions must meet. It specifies principles and criteria for key stages such as publication, discovery, negotiation, exchange, and usage. The previous CWA documents provided the initial input to the CEN/CLC/JTC 25 Joint Technical Committee - Data management, Dataspaces, Cloud and Edge,^28^ which is leading the development of the future harmonized standard Trusted Data Transaction, along with the ETSI standardization organization. The future standard will support the implementation of Article 33 of the EU’s Data Act, which addresses essential requirements for interoperability of data, data sharing mechanisms, and services, as well as common European Data Spaces. JTC 25 is also working on a standard to assess the maturity of data spaces, based on the DSSC maturity model [24].

*ETSI* ETSI is a European standardization body specializing in ICT, network, and security standards supporting the EU’s digital single market. Regarding data spaces, in the beginning of 2025, ETSI has launched a new Technical Committee on Data Solutions (TC DATA) aimed at advancing data-driven technologies and supporting the development of new industry standards. ETSI TR 104 409 V1.1.1 [25] is one of its first publications and it is devoted to analysing the requirements established by Article 33 of the EU’s Data Act and collecting a set of references that could be relevant to fulfil those requirements.

*ISO/IEC* ISO/IEC is other standardization body that is addressing data spaces normalization, having already published the ISO/IEC Draft International Standard (DIS) 20151 [26]. This standard aims to define foundation concepts, key characteristics, and essential requirements for data spaces, including definitions, interoperability baselines, and the distinguishing criteria between data spaces and related constructs such as data lakes.

## 3 Digital Identity Ecosystem

This Section summarizes the identity and credential management landscape including main concepts and technologies, the European Strategy for Digital Identity, and the most relevant organizations and initiatives contributing to the standardization of self-sovereign identity.

### 3.1 Concepts, models and technologies

In this section, key concepts, models, and technologies related to digital identity are introduced, which will be referenced throughout the work. Since these terms have been described in multiple ways across the literature, concise definitions are adopted here to capture their most relevant meaning within the scope of this study.

An *entity* [27,28] is a physical or logical object with independent existence that can be reference such as people, organizations, concepts, etc. An *attribute* [29] expresses a characteristic of an entity, defined within a context and whose value describes or distinguishes the entity among others. An *identifier* [30] is a carefully chosen attribute of an entity whose value is intended to uniquely distinguish that entity within a specific context or domain. Note that some sources consider that an identifier can be a set of more than one attribute, given that they uniquely identify an entity within a context. A *(digital) identity* [27] is then defined as the digital representation of a unique identifier, associated with the set of all the attributes of an entity within a given context and policies. A *profile* [30] is a subset of identity attributes shared in a specific context, enabling controlled information exchange across different services or domains.

**Fig. 3:**
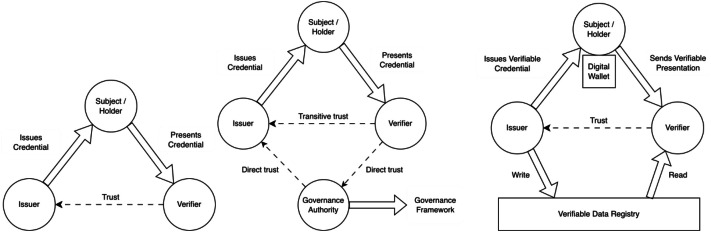
Evolution of the credential trust model: (a) The credential trust triangle. Adapted from [35]. (b) The governance trust diamond. Adapted from [35]. (c) The verifiable credential trust triangle in the decentralized identity model. Adapted from [35].

In digital systems (and in physical ones), an entity typically must prove certain attributes to access a resource. Credentials are the default mechanism for this purpose. A *subject* [31,32] is an entity that receives credentials from an issuer and is described by the claims associated with it. A *claim* (or *attestation*) [33,28] is an assertion about a subject, typically made by an issuer as part of a credential. A *credential* [28] is a collection of claims issued by an entity, linked to an identifier, that provides information about one subject (or more than one). An *issuer* [5,4] is an authorized entity that generates credentials about a subject, attests to their validity, and transmits the credential to a holder. A *holder* [33,28] is an entity that possesses one or more credentials, often on behalf of a subject, and may present them to a verifier. The holder stores the credentials in a *wallet*. A *verifier* [28] is an entity that receives credentials or claims, typically from a holder, and checks their validity before granting access or performing a specific action. The credential trust triangle illustrates how credentials are used and why: the trust the verifier places in the issuer enables the verifier to trust the credential attesting certain attributes of a subject (see Fig. 3a) [34].

Often, several entities may act as issuers of a certain type of credential, which poses scalability challenges. The governance trust diamond (see Fig. 3b) introduces a fourth entity: the *governance authority*. This entity develops and publishes a governance framework that documents the policies and rules for a specific trust community. In order to be directly trusted as issuers by the governance authority, entities must prove compliance with the governance framework. If verifiers trust the governance authority and the governance framework, they are also (transitively) trusting the issuers.

In the last decades, identity management models have evolved from centralized identity models to decentralized identity models, reflecting a shift toward greater user autonomy and control over personal data [36,37]. In decentralized identity (DI) models, identities are managed without a central authority, relying instead on Verifiable Data Registries. A *Verifiable Data Registry* [28] is a trusted system that stores and enables verification of identifiers, cryptographic material, credential schemas, and revocation lists; they are usually implemented with distributed technologies such as blockchain or distributed ledgers. Entities hold cryptographic keys that allow them to create, control, and share their digital identifiers directly. Other entities can verify these identifiers through decentralized networks (e.g., Verifiable Data Registries), eliminating single points of failure and reducing dependency on traditional identity providers.

Decentralized identity models are also known as Self-Sovereign Identity (SSI) models, emphasizing that individuals own and govern their digital identities without reliance on a single central authority [36]. In addition, SSI models allow users to decide what information want to share, with whom, and under what conditions. Unlike federated identity systems, SSI puts the user at the center, ensuring transparency, privacy, and autonomy in the storage and exchange of identity data. SSI is enabled by distributed infrastructure like Verifiable Data Registries and a set of technologies such as Decentralized Identifiers (DIDs), Verifiable Credentials (VC), Verifiable Presentations (VP) and Digital Wallets.

*Decentralized Identifiers (DIDs)* [38] are a type of identifier that enables verifiable and decentralized digital identity. Although third parties can facilitate the discovery of information associated with a DID, the DID controller retains full cryptographic control and can demonstrate ownership without needing authorization from anyone else. DIDs are represented as URIs and connected to DID Documents, which enable secure and verifiable interactions with the associated DID subject [39]. On the other hand, DIDs Documents are a structured data objects (typically expressed in JSON) which contain information about a DID subject, such as public keys, verification methods, and service end points. DID Methods define how a DID is created, resolved, updated, and revoked on a specific distributed ledger or blockchain [38,39].

A *Verifiable Credential (VC)* [28] is a credential designed to resist tampering, whose origin and integrity can be confirmed through cryptographic methods. It can serve as the basis for creating verifiable presentations. On the other hand, a *Verifiable Presentation (VP)* [28] is a tamper-resistant package of information that allows the receiver to trust its origin and content through cryptographic verification. It may include original credentials or derived proofs, such as zero-knowledge proofs. *Digital Wallets*, in the context of SSI are a secure software tool, often running on a personal device or in the cloud, that enables users to store and manage their identity credentials, cryptographic keys, and other sensitive information (notice that credential storage systems receive other names across different sources). It provides full control over personal data, supporting key generation, secure backup, credential issuance, and verification. By using a digital wallet, users can interact with third parties in a trusted manner, sign transactions or claims, and maintain data portability without relying on centralized storage, ensuring that identity information remains under the user’s control [40].

The verifiable credential trust model enables trust between the verifier and the issuer using the Verifiable Data Registry as an intermediary. Fig. 3c shows the VDR acting as a trusted store for the issuer’s public key. Verifiers retrieve this cryptographic material when needed to verify a verifiable presentation from a holder who possesses a verifiable credential issued by that issuer.

**Fig. 4:**
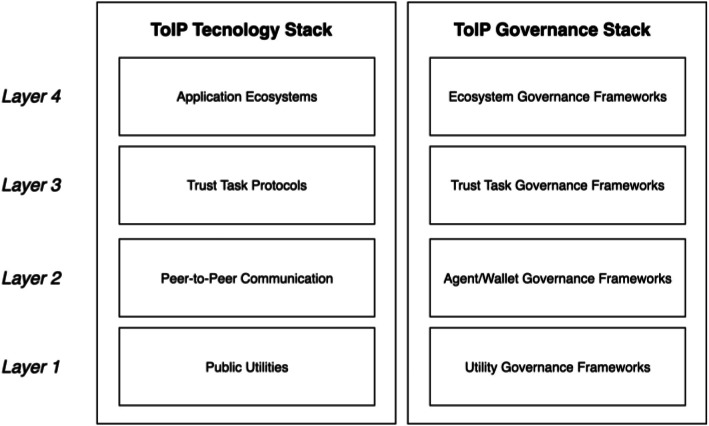
Conceptual model of the Trust Over IP (ToIP) Stack. Adapted from [41].

The verifiable credential trust triangle supported by the decentralized identity model has been taken by the Trust Over IP Foundation^29^ as the basis to define an overall architecture for Internet-scale digital trust in the form of a double-sided four-layer stack [35,41]. Fig. 4 shows a minimal representation of the ToIP Stack. While the left side of the stack addresses the technological aspects, the right side focuses on the governance ones in each of the layers. Each layer takes charge of different responsibilities:

• Layer 1 is for the public DID utilities (e.g., DID definition, DID methods) that are needed to look up and verify the current public keys of issuers of digital credentials. It is also known as the trust support layer because it takes in the functions designed to specifically support machine-to machine trust like identity binding mechanisms that associate a natural person to an agent and hardware-based trust attestation systems. Supporting systems, like decentralized record-keeping technologies, are often associated to this layer but can be used by any element from any layer when needed.
• Layer 2 is the trust spanning layer of the ToIP stack. This layer focuses on the digital wallets and agents needed by individuals, organizations, and other digital entities, and how they interact to establish secure connections based on both parties’ DIDs over a standard peer-to-peer protocol.
• Layer 3 manages additional trust task functions built upon the trusted peer-to-peer communication layer provided by Layer 2. It supports the implementation of the verifiable credential trust triangle, enabling the establishment of transitive trust relationships among three parties using verifiable credential data exchange formats and protocols.
• Layer 4 is an open-ended application layer for any application that requires trusted interactions.

### 3.2 European strategy for digital identity

The European Strategy for Digital Identity centers on the EU Digital Identity Framework (also Known as eIDAS. 2.0), which aims to provide a secure, user-friendly, and unified digital identity solution for citizens, businesses, and residents across the EU by 2030. Next the most important European regulations (eIDAS and EUDI Wallet) are described, together with the EU funded initiative European Blockchain Services Infrastructure (EBSI).

*eIDAS 2.0* In Spain, since July 1, 2016, eIDAS, Regulation (EU) No. 910/2014 of the European Parliament and of the Council, of July 23, 2014, on electronic identification and trust services for electronic transactions in the internal market [42] repealed Directive 1999/93/EC.

The eIDAS Regulation establishes a unified legal framework across the EU for electronic identification (eID) and trust services in electronic transactions. It ensures mutual recognition of national eID systems for accessing public services across borders and provides legal certainty for trust services such as electronic signatures, seals, timestamps, registered delivery services, and website authentication. The Regulation defines levels of assurance for eID, distinguishes between qualified and non-qualified trust services, and sets rules for supervision, liability, and security requirements. Its aim is to build trust in the digital single market by enabling secure and seamless electronic interactions between businesses, citizens, and public authorities throughout the EU. The 2024 amendment to the eIDAS Regulation (Regulation (EU) 2024/1183) [43], known as eIDAS 2.0, establishes a comprehensive framework for the European Digital Identity (EUDI) Wallet, expanding and modernizing the 2014 regulation (EU No 910/2014). Its aim is to provide all EU citizens and residents with a secure, user-controlled, and widely accepted digital identity, facilitating access to both public and private services across the Union.

*EUDI Wallet*^30^ The European Digital Identity (EUDI) Wallet, regulated in [42], allows users to store, manage, and share identification data and electronic attestations of attributes, such as diplomas or licenses, securely and selectively. It supports qualified electronic signatures and seals, recognized across the EU, and must operate under high security and privacy standards, including data minimization, user control, and transparency.

The regulation mandates interoperability, non-discrimination (users must not be forced to use the Wallet), and free access for non-professional use. It integrates with other EU frameworks (like the GDPR and the Cybersecurity Act) and allows additional features (e.g., pseudonyms, offline use, and dashboards for transaction tracking). Certification and oversight mechanisms are defined, along with rules for trust service providers offering electronic archiving, electronic ledgers, or attestation of attributes. In short, this amendment creates the legal and technical backbone for a unified digital identity system in Europe, aiming to boost digital inclusion, trust, cybersecurity, and cross-border service access.

Six large-scale pilots have been launched to test the EUDI Wallet before rolling it out in the EU Member States. While four of them are already closed, two remain still active.^31^ It is expected that at the end of 2026 each country must provide at least one operational EUDI Wallet.

*European Blockchain Services Infrastructure*^32^ *(EBSI)* The European Blockchain Services Infrastructure (EBSI) is a cross-border, Europe-wide blockchain backbone designed to enable interoperable public services and trusted digital interactions across EU member states. One of the main contributions of EBSI is its Verifiable Credentials Framework. It implements the previously mentioned credential trust triangle supported by a Verifiable Data Registry implemented through the EBSI blockchain. EBSI has defined its own DID Method for legal entities ‘did:ebsi’ and a profile of the W3C’s^33^ Verifiable Credential Data Model v2.0 [28]. The blockchain stores DID Documents of the Trusted Issuers (TI) —which in turn are accredited to be so by the Trusted Accreditation Organisations (TAO) and these by the Root Trusted Accreditation Organisation (Root TAO)— and the schemas that define the verifiable credentials models.

Other standards used in the EBSI’s Verifiable Credentials Framework are Open ID for Verifiable Credentials Issuance (OID4VCI) [44] and Open ID for Verifiable Presentations (OID4VP) [45] —both by the W3C—, along with the DIF’s Presentation Exchange 2.0.0 protocol [46] and the W3C’s Bitstring Status List v1.0 [47]. Note that the standards mentioned herein will be described next, in Section 3.3.

### 3.3 Organizations and standards

This Section summarizes the most relevant organizations supporting standardization and adoption of digital identity technologies.

*World Wide Web Consortium (W3C)* The World Wide Web Consortium (W3C) is an international community dedicated to developing open standards for the Web. In the self-sovereign identity space, the W3C has published key specifications for verifiable credentials and decentralized identifiers. The Verifiable Credentials Data Model v2.0 (VCDM) [28] specifies an extensible data model for expressing cryptographically secure, privacy-respecting, and machine-verifiable digital credentials and presentations, enabling trusted claims exchange between issuers, holders, and verifiers in web-based identity ecosystems. VCDM 2.0 is one of the most popular verifiable credential models. Another W3C’s key contribution in the self-sovereign identity landscape is the Decentralized Identifiers (DIDs) v1.0 recommendation [48], that specifies a data model and syntax for decentralized, globally unique identifiers that do not require a centralized authority, enabling entities to create, control, and cryptographically prove ownership of their identifiers.

Other critical specifications published by the W3C are the DID Methods Note [49], that lists the known DID methods in the decentralized identity ecosystem, and the recommendation JSON-LD 1.1 [50], that allows to extend the VCDM 2.0 —by adding customized semantic context—, and supports Linked Data Proofs —also standardized by the W3C in the Verifiable Credential Data Integrity specification [51]—.

*Decentralized Identity Foundation*^34^ *(DIF)* The Decentralized Identity Foundation (DIF) is an engineering focused consortium committed to developing open standards, technology, and libraries for decentralized identity ecosystems. DIF mainly contributes to interoperable, scalable decentralized identity architectures, focusing on identifiers, verifiable credentials, authentication, and protocols enabling privacy and trust.

One of DIF’s most important contributions in the self-sovereign identity landscape is the DIDComm Messaging specification.^35^ DIDComm Messaging specifies a secure, privacy-preserving transport protocol for exchanging messages between endpoint peers with Decentralized Identifiers (DIDs).

On top of the DIDComm Messaging protocol, DIF is standardizing the Credential Manifest data model,^36^ enabling issuers to express the types of verifiable credentials they can issue along with the input requirements, and the Presentation Exchange data model and protocol [46], enabling verifiers to describe and communicate credential presentation requirements to credential holders. Furthermore, DIF has also standardized two protocols for the issuance of verifiable credentials, the Issue Credential Protocol 3.0,^37^ and the exchange of verifiable presentations, the Present Proof Protocol 3.0.^38^

*OpenID Foundation*^39^ The OpenID Foundation is a non-profit international standardization organization focused on digital identity and authentication. OpenID Foundation develops standards for federated identity and strong authentication, now expanding to decentralized identity and trust frameworks, making crucial contributions to identity interoperability in the EU context. One of the most well-known contributions of the OpenID Foundation to the self-sovereign identity landscape is the OpenID Connect^40^ (OIDC) protocol suite which provides an identity authentication layer built on top of OAuth 2.0 authorization protocol [52].

The OpenID Foundation is also working on specifications that focus on enabling the credential trust triangle, being the most well-known the Self-Issued OpenID Provider v2 (SIOPv2) draft specification [53], that allows entities to use an identity provider under their control in OpenID Connect, and the pair of specifications supporting the issuance of verifiable credentials — OpenID for Verifiable Credential Issuance 1.0 (OID4VCI) [44]— and its presentation — OpenID for Verifiable Presentations 1.0 (OID4VP) [45]—.

*Eclipse Foundation* The Eclipse Foundation also plays a role in the self-sovereign identity (SSI) landscape with several initiatives, being the most relevant for this work the well-known the Eclipse Decentralized Claims Protocol (DCP) v1.0-RC4 [21]. This specification defines a set of protocols for asserting participant identities, issuing verifiable credentials, and presenting verifiable credentials using a decentralized architecture for verification and trust.

*IETF* The Internet Engineering Task Force (IETF) is the global standards organization for Internet protocols and architectures. IETF’s work directly supports secure communication, identity, and credential exchange through rigorous and interoperable standards such as the OAuth 2.0 Authorization Framework [52] and the Selective Disclosure JWT based Verifiable Credentials (SD-JWT VC) Internet Draft [54] that describes data formats as well as validation and processing rules to express Verifiable Credentials with JSON payloads with and without selective disclosure.

*Hyperledger Foundation*^41^ Hyperledger Foundation, hosted by the Linux Foundation and now expanded in the Linux Foundation Decentralized Trust (LFDT), is an open-source collaborative group focused on developing enterprise-grade distributed ledger technologies and tools. Hyperledger projects address decentralized identity, verifiable credentials, self-sovereign identity, and trust models suited for cross-border data spaces and EU regulatory settings. The most relevant projects developed within the Hyperledger Foundation are the Hyperledger Fabric blockchain framework^42^ and the Hyperledger Indy distributed ledger,^43^ specifically designed to create and use decentralized identities.

Another relevant contribution of the Hyperledger Foundation to the self-sovereign identity landscape is the Hyperledger AnonCreds project,^44^ that is working on the specification to implement the credential trust triangle (recall Fig. 3) using anonymous credentials [55].

*Trust over IP Foundation* The Trust Over IP Foundation (ToIP), also part of the Linux Foundation, builds open standards and governance for digital trust interoperability. ToIP’s focus is on multi-layered trust architectures, self-sovereign identity adoption, and frameworks that connect technical, legal, and organizational trust mechanisms worldwide. Besides the double-sided four layer Trust over IP (ToIP) Stack model (recall Fig. 4), ToIP has published several relevant technical specifications such as the Trust Spanning Protocol (TSP) [56] that specifies a universal, message-based protocol to enable secure, privacy-preserving communication between endpoints that may use different types of verifiable identifiers, and the Trust Registry Query Protocol (TRQP) V2.0 [57] that enables fast and efficient queries for authoritative data from trust registries, enabling participants in a self-sovereign identity system to verify whether entities hold specific authorizations or recognitions under a certain governance framework.

*ETSI* ETSI has supported the EU in the definition and deployment of the EU’s vision of digital identity in the last decades. ETSI is currently developing new specifications related to the EU’s vision of self-sovereign identity, being the most relevant for this work the ETSI TS 119 612 V2.4.1 [58]. This standard specifies the format, mechanisms, and procedures for establishing, accessing, and authenticating trusted lists that contain information about qualified trust service providers (TSPs) and their services in compliance with EU regulations such as eIDAS. In the context of self-sovereign identity, this standard enables the formal recognition and verification of trust service providers and their credentials, facilitating the validation of digital signatures, certificates, and identity attributes issued or vouched for by these providers.

*ISO/IEC* The ISO/IEC (International Organization for Standardization/International Electrotechnical Commission) is a worldwide federation developing international standards for the areas of information technology, cybersecurity, and identity, among others. The ISO/IEC 18013 group of standards is relevant to the self-sovereign identity landscape as it outlines the technical and operational requirements for physical and mobile driving licenses. Particularly, the ISO/IEC 18013-5 [59] defines the specifications for mobile driving licenses (mDLs) and mobile credentials, specifying secure interfaces and data formats that enable identity verification and management primarily in in-person interaction scenarios.

## 4 Identity and Credential Management in Gaia-X

### 4.1 The need to bridge the gap between data spaces and self-sovereign identity ecosystems

Although data spaces and self-sovereign identity (SSI) domains share aligned goals, a gap remains between them, and bridging this gap is essential to effectively address the self-sovereign data sharing challenge. The DSSC Blueprint v2.0 [12] defines several technical building blocks focused on identity, credentials and trust management. The Blueprint identifies verifiable credentials and trust entities (e.g., Trust Anchors and Trust Service Providers) as key identity and trust components for constructing SSI-enabled data spaces. However, as a reference architecture, it does not prescribe any particular technology for its implementation.

On the other hand, SSI technologies and standards remain fragmented, with some overlap, and the ToIP Stack continues to evolve and mature. This situation is likely to hinder the development of data spaces. Therefore, data spaces will benefit from explicit guidelines and frameworks that help specify how identity and trust are managed across diverse organizations that want to share data, ensuring security, federated governance, and interoperability. In the European context, iSHARE^45^ and Gaia-X emerge as the two primary frameworks addressing these needs. Although their functionalities overlap regarding identity and trust management, their operational logic for data access differs: iSHARE relies on a registry-based delegation model where authorizations are stored by an Authorization Registry. In contrast, Gaia-X adopts a decentralized policy-enforcement approach, where access is granted by validating a consumer's Verifiable Credentials against the specific policies associated with a data offering. This latter approach, combined with Gaia-X’s rigorous, automated compliance framework for infrastructure, positions it as a more comprehensive and systemic solution for end-to-end trust management in complex data spaces.

### 4.2 Gaia-X framework and core services

GAIA-X is a European initiative that aims to create a secure, federated, and interoperable data infrastructure. It provides a common framework that allows organizations to share data and digital services while retaining control over their assets. It promotes openness and trust by defining rules, standards, and technical building blocks that ensure data usage is lawful, traceable, and aligned with European values such as privacy, data sovereignty, and fair competition. As described in Section 2.5, key contributions of Gaia-X are the Trust Framework, the Digital Clearing House and the Labels.

Several specifications describe how Gaia-X's Trust Framework works, stating functional and technical requirements:

• The Gaia-X's Architecture document [60] sets technical and organizational standards for onboarding participants and verifying compliance against a set of rules.
• On the other hand, the Gaia-X's Compliance document [61] defines criteria that services and participants must fulfil to be able to participate in a Gaia-X compliant data space.
• Gaia-X's Identity, Credentials and Access Management (ICAM) document [62] specifies the essential components for authentication, authorisation, and access management for Gaia-X compliant data spaces and introduces the concepts of decentralized identifiers, verifiable credentials, verifiable presentations, and credentials’ trust scope. The ICAM specification also defines the accepted identifiers, the credential formats and their semantic model, via a specific extension of the W3C Verifiable Credential Data Model 2.0 [28] with the Gaia-X Ontology.
• Finally, the Gaia-X's Data Exchange document [63] specifies the set of services that provides features enabling data exchanges and delves into the concept of data product –a collection of one or more data ready for the data exchange—, including a high level architecture and key requirements for data value, trust and compliance.

**Fig. 5:**
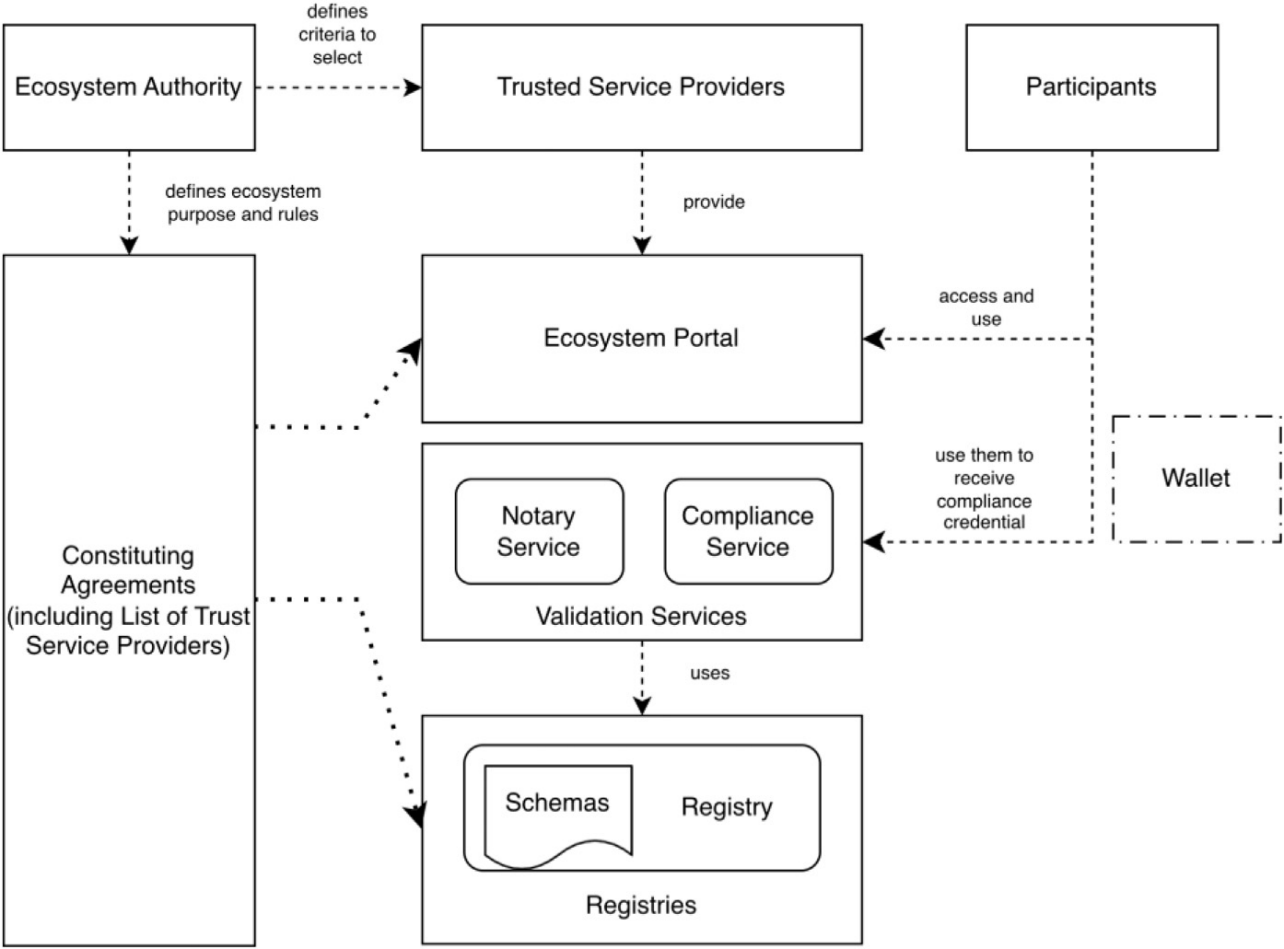
Conceptual model of the Gaia-X architecture. Adapted from [60].

Another central element in Gaia-X is the Digital Clearing House (GXDCH). Digital Clearing Houses are decentralized, independently operated nodes responsible for verifying compliance, and providing essential and optional services. The architecture is supported by open-source reference implementations and a Technical Compatibility Kit (TCK). The essential core services –currently considered as mandatory for Digital Clearing Houses– are the following ones (see Fig. 5):

• The Gaia-X Registry acts as a decentralized repository of key ecosystem information, including Trust Anchors, compliance rules, and credential schemas.
• The Gaia-X Notary Service converts non-machine-readable proofs into Gaia-X verifiable credentials.
• The Gaia-X Compliance Service validates the credentials of participants and service offerings by verifying their compliance with Gaia-X rules and criteria. It issues a verifiable credential that attests to the result of this validation and can be stored in the wallet of the participant.

In the Loire release of the Gaia-X Architecture, Trust Service Providers issue verifiable credentials for a given scope and purpose, while Trusted Data Sources are the source of the information used by a credential issuer to validate attestations [60]. Key examples of Trust Service Providers include Trusted Identity Providers (e.g., entities issuing Qualified eIDAS Certificates and EV SSL Certificate Issuers); an example of Trusted Data Source is the Global Legal Entity Identifier (GLEIF) API. Currently governance and trust are anchored by the Gaia-X Association, which defines policies in the Compliance document, manages Trust Anchors through the Registry Service, approves GXDCH operators that oversee that participants and services are compliant with Gaia-X rules. All this ensures that participants exchange data and services in line with privacy, security, and European digital sovereignty principles. However, the true potential of Gaia-X for a federated and secure trust management lies in the future framework’s inclusion of customizable Trust Anchors and personalized Compliance rules by the Ecosystem Authority. The next Gaia-X framework release (Danube) is expected to introduce such mechanisms.

### 4.3 Gaia-X's associations and collaborations

*Alignment with Foundations and Associations* Gaia-X aligns with key European associations that support the development of data spaces. Notably, it works with the International Data Spaces Association (IDSA) on the Dataspace Protocol and contributes jointly to the draft ISO/IEC 20151 standard. It also collaborates with the FIWARE Foundation and the Big Data Value Association (BDVA).

*Cooperation with External Projects and Initiatives* Beyond its alliances, Gaia-X is also embedded in broader European projects. It participates in the DSSC project, contributing to the DSSC Blueprint and engaging with communities of practice to accelerate data space adoption across sectors. The association also explores synergies with SIMPL, aligning within Gaia-X technical and business committees, and collaborates with the DOME project, which seeks to harmonize compliance frameworks for marketplaces and integrate Gaia-X clearing house mechanisms. Additionally, Gaia-X and iSHARE are working together to achieve a minimum technical compatibility. These engagements illustrate Gaia-X’s role not only as a standards-setter but also as an active contributor to pilot projects and experimental platforms.

*Engagement with Standards and Regulations* The credibility of the Gaia-X Framework derives from its alignment with European regulations and international standards. It complements eIDAS and eIDAS 2.0, mapping credential services to European Digital Identity Wallets like the EUDI Wallet. Gaia-X also contributes to CEN/CENELEC pre-standardization and the Eclipse Dataspace Working Group. Finally, Gaia-X’s approach is coherent with key EU legislation, including GDPR, the Data Act, and the Data Governance Act, ensuring privacy, security, and digital sovereignty.

### 4.4 Mapping Gaia-X to the DSSC blueprint and the ToIP stack

Gaia-X primarily targets the DSSC's Identity and Attestation Management and the Trust Framework Building Blocks. It also contributes to the Participation Management building block, since the underlying technical components underpin governance and enable cross-domain participation. Although the specifications of the two organisations—terms, components, and their relations—are not yet fully aligned, they show clear convergence with each update and refinement of their documents. In Table 1 we present the result of analysing both proposals and the match between the DSSC’s building blocks and services and the Gaia-X's main components.

**Table 1:** Positioning Gaia-X in the DSSC Blueprint v2.0 by matching Gaia-X services with the relevant DSSC technical building blocks and services.

DSSC’s Technical Building Blocks	DSSC’s Services for Implementing Technical Building Blocks				
	Federation Services				Participant Agent Services
	Data Space Registry	Policy Information Point		Validation and Verification	Credential Store
		Identity Provisioning	Conformity Assessment		
Identity and Attestation Management	X	X	X	X	X
Trust Framework	X	X	X	—	—
Gaia-X Services / *Services used in Gaia-X Trust Framework*	Gaia-X Registry	*Trust Service Providers and Trusted Data Sources*		Gaia-X Notary and Compliance Services	*Wallet*

On the other hand, the ToIP Stack is less familiar –and less frequently cited– within the data spaces domain, including Gaia-X. The relationship between the two can be clarified by identifying which SSI technologies and standards are used in data spaces or data spaces’ technologies and frameworks. The latest Gaia-X Architecture document [60] lists supported SSI technologies that help to establish the relationship between Gaia-X and the ToIP Stack.

Concerning Layer 1 of the ToIP Stack, the Loire release of Gaia-X only supports only the ‘did:web’ method; while the Danube release is expected to support ‘did:web’ and ‘did:key’ methods [49], with additional methods available via extensions. The Trust Anchors recognized by Gaia-X (and listed in the Gaia-X Registry) are also part of this layer, and compatible with the concept of Trust Service Providers and Authentic Sources defined in the EUDI Architecture and Reference Framework.^46^

For Layer 3, Gaia-X relies on the W3C Verifiable Credential Data Model (VCDM) v2.0 [28] and the W3C VC Bitstring Status List [47]. Both the Loire and Danube releases support JSON Web Token (JWT) Verifiable Credentials [54] and the OpenID Connect protocol suite for Verifiable Credentials (OID4VCI [44] and OID4VP [45]) to securely exchange credentials. The EUDI Architecture and Reference Framework also recognizes the OID4VCI and OID4VP protocols for issuing and presenting credentials remotely, facilitating the compatibility of Gaia-X and EUDI Wallets.

Layer 2 in Gaia-X Trust Framework currently relies on the OpenID Connect (OIDC) protocol suite and OAuth2.0 [52]. As with the DID Methods, Gaia-X expects to support additional Layer 2 and 3 protocols and data models via extensions. For Layer 4, Gaia-X Architecture document [60] establishes the alignment of the Trust Framework with the Eclipse Dataspace Protocol [20].

From the analysis, Gaia‑X emerges as a trust framework for federated data spaces, encompassing Layers 1 to 3 of the ToIP Stack. Currently, Gaia-X supports a specific set of identity protocols and technologies, but it has already stated its intention to pursue interoperability with additional ones.

### 4.5 Presence of Gaia-X in the Data Spaces Radar

**Fig. 6:**
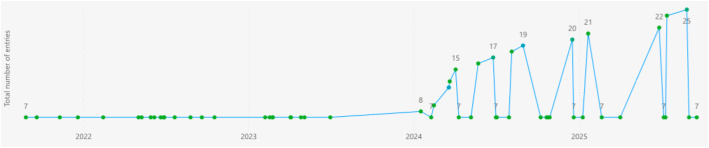
Evolution of the number of data spaces that follow the Gaia-X architecture as registered in the Data Spaces Radar tool.

To conclude this section, we have used the Data Spaces Radar tool^47^ to get an overview of the presence of Gaia-X in the Data Space registered. At the end of the first semester of 2025 around 25 Gaia-X aligned Data Space projects and use cases (see Fig. 6) were registered in the Radar, constituting a little more than the 10% of the total amount of cases registered. The 16% of the Gaia-X cases are in the operational development stage (compared to the 11% of the total cases), 64% under implementation (versus the 54% of the total cases) and the remaining 16% in the preparatory phase (compared to the 25% of the total cases). Most of the Gaia-X aligned projects declare to have started during 2024 or 2025. This is aligned with the availability of funding opportunities emerged from the European Data Strategy 2020.

## 5 Systematic Literature Review

In this section we present the Systematic Literature Review done with the purpose of analyzing how literature on data spaces aligned with the Gaia-X framework address identity and credentials management.

### 5.1 Methodology

**Fig. 7:**
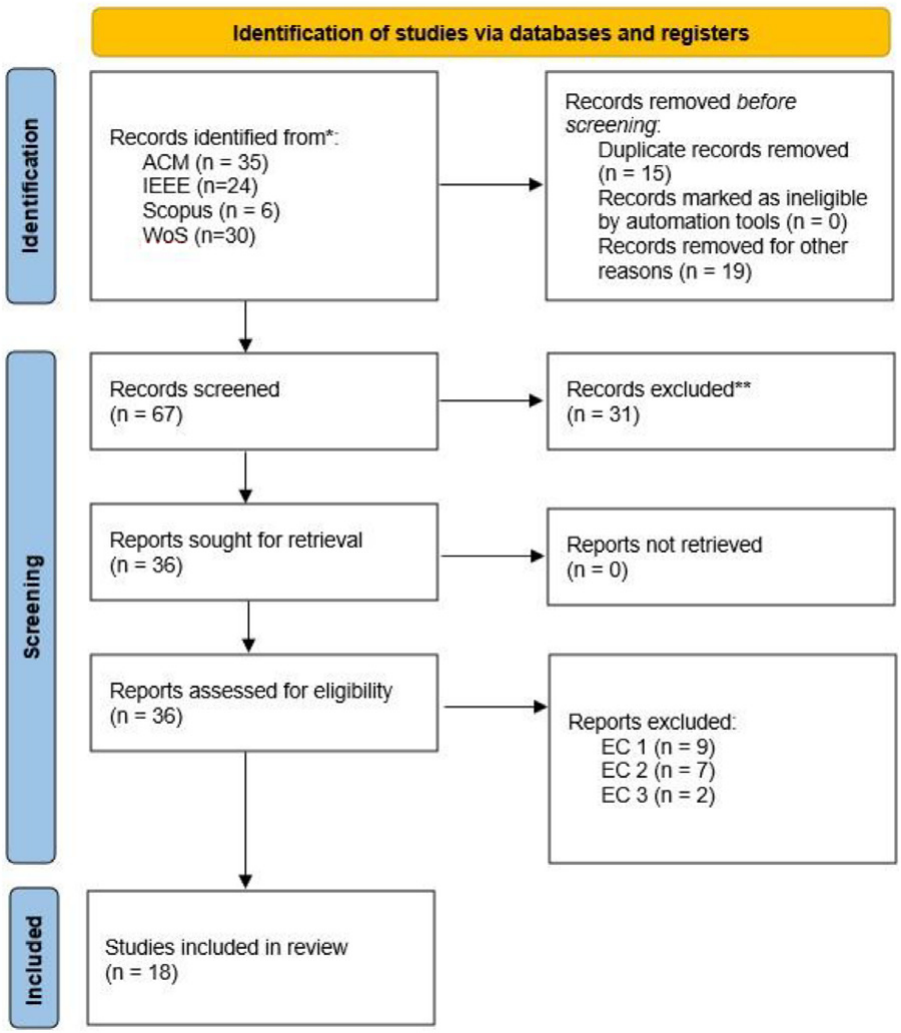
PRISMA flow diagram illustrating the selection process for the systematic literature review on data spaces.

We conducted a systematic literature review following the PRISMA (Preferred Reporting Items for Systematic Reviews and Meta-Analyses) methodology [64]. This approach structures the review process into four phases: identification, screening, eligibility, and inclusion. Searches were performed across major scientific databases (Scopus, IEEE Xplore, ACM, Web of Science) using specific keywords. The whole process is illustrated in Fig. 7.

### 5.2 Research questions and exclusion/inclusion criteria

The following questions have been identified as key elements to structure the literature review:

• **Q1: Architecture.** How do the reviewed works define the overall architecture of the data space? Do they explicitly include the connector, specify which type of connector is used, and indicate whether they follow reference architectures such as Gaia-X, IDSA, DSSC or other federated data space models?
• **Q2: Identity and Credential Governance.** How is trust established and managed in identity and credential systems? Do the works mention Wallets together with verifiable credentials (VCs) and verifiable presentations (VPs)? Do they use Distributed Ledger Technologies as trust anchors? The answer to these questions helps to understand the credential trust model underlying the published proposals even if it is not explicitly specified.
• **Q3: Identity and Credentials Technologies and Standards.** Which technological stacks, protocols, and infrastructures are adopted to implement authentication and credential exchange? The answer to this question helps understand the alignment of the proposal with the ToIP Stack.
• **Q4: Compliance** Which regulatory frameworks (e.g., GDPR, eIDAS/eIDAS2) are considered in the proposed solutions?
• **Q5: Evaluation and Maturity.** What is the reported level of maturity of the solutions (e.g., conceptual model, proof-of-concept, pilot, production)?
• **Q6: Implementation Challenges and Open Issues.** What implementation challenges are reported in the literature (e.g., performance bottlenecks, fragmented standards, limited ecosystem alignment)? Which unresolved issues hinder the adoption and scalability of authentication mechanisms in data spaces?

To ensure the relevance and focus of the review, we applied the following exclusion and inclusion criteria when selecting publications:

**Exclusion criteria:**

• **EC-1:** the publication does not discuss data spaces.
• **EC-2:** the publication does not include information about credentials.
• **EC-3:** the publication is a systematic literature review or a survey.

**Inclusion criteria:**

• **IC:** the publication addresses identity and credentials in data spaces.

### 5.3 Identification

In this stage, a comprehensive search was conducted across selected databases and other relevant sources to identify potentially eligible studies. The aim was to capture a broad and inclusive set of records related to the research topic.

Keywords and search strings were carefully designed to reflect the scope of the study. Table 2 presents the specific queries used to search potentially relevant works in the four databases used. Our justification for selecting this set of search terms in the queries rests on two core ideas: first, to identify works related to the Gaia-X framework we included the term “gaia-x” but, recognizing Gaia-X’s recent emergence, we also incorporated a broader alternative using an OR with the term “data space” to balance specificity and coverage; second, to capture relevant discussions on verifiable credentials within a European context we searched for “verifiable credentials” while also including “EUDI” and “identity wallet” to surface implementations and discussions aligned with the European scenario. After performing the search, duplicate entries were subsequently removed to ensure accuracy in the following screening phase. A total of 67 records were removed prior to screening: 15 were duplicates, and 19 were excluded for other reasons, 2 because a more recent version of the same work was available, and 17 because they corresponded to entire conference proceedings rather than individual studies. The remaining records proceeded to the screening phase.

**Table 2:** Queries used to search the databases.

Database	Search	Papers found
ACM	[[All: "gaia-x"] OR [All: "data space"]] AND [[All: "verifiable credential"] OR [All: "eudi"] OR [All: "identity wallet"]]	35
IEEE Explore	("All Metadata":GAIA-X OR "All Metadata":data space) AND ("All Metadata":verifiable credential OR "All Metadata":EUDI OR "All Metadata":identity wallet)	24 (3 open access)
Scopus	(TITLE-ABS-KEY (gaia-x OR "data space") AND TITLE-ABS-KEY ("verifiable credential" OR eudi OR "identity wallet"))	6
Web of science	(ALL=(gaia-x) OR ALL=(data space)) AND (ALL=(verifiable credential) OR ALL=(eudi) OR ALL=(identity wallet))	30

### 5.4 Screening

Titles and abstracts of the remaining records were reviewed based on the previously defined guiding questions. This initial assessment led to the exclusion of 31 records that did not meet the inclusion criteria, leaving 36 studies for full-text review.

All of the 36 studies selected for full-text review were successfully retrieved.

### 5.5 Assessed for eligibility

A total of 36 full-text articles were assessed for eligibility. Based on the exclusion criteria, 16 were removed:

• **EC-1**: 9 articles were excluded
• **EC-2**: 7 articles were excluded
• **EC-3**: 2 articles were excluded

After applying these criteria, 18 studies compliant with **IC** remained for the inclusion step.

### 5.6 Results

**Fig. 8:**
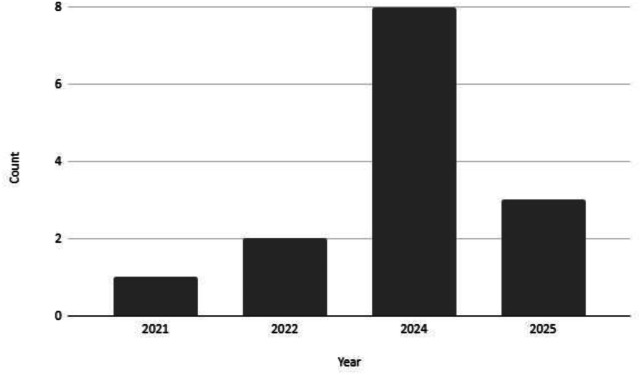
Number of articles per year on authentication and data spaces.

The number of publications addressing identity, credentials, and authentication in data spaces has steadily increased in recent years, reflecting the growing importance of these topics in the context of initiatives such as Gaia-X (see Fig. 8). Next, we analyse the selected publications (see Table 3) to answer the questions we identified in Section 5.2.

**Table 3:** Summary of the results of the systematic literature review.

Article (Year)	Q1: Architecture	Q2: Identity and credential governance	Q3: Identity and credentials Technologies and Standards	Q4: Compliance	Q5: Evaluation and Maturity	Q6: Implementation Challenges and Open Issues
[65] (2021)	SSI (eIDAS)	X.509 certificate	OAuth2.0, SAML 2.0, JWT, AuthZ	eIDAS	Proof of concept with 30 users	Identification and Identity Governance
[66] (2022)	SSI (eIDAS)	Wallet, VCs	UMA OAuth 2.0, SAML2	GDPR, eIDAS and eIDs	Use case	Identification and Identity Governance
[67] (2022)	GAIA-X	VCs	JSON RDF, XML	Not mentioned	Concept description	Validation in Real-World Environments + Interoperability and Architecture Extension
[32] (2023)	GAIA-X, EDC	VCs	OIDC, W3C JSON-LD, TRAIN, UCON+	Beyond scope of paper	Use case and proof of concept	Interoperability and Architecture Extension
[68] (2023)	GAIA-X	VCs, DLT	OIDC, Hyperledger ARIES INDY AnonCreds, walt.id, keycloack IAM broker, SIOPv2, IOTA	eIDAS	Use case	Interoperability and Architecture Extension
[69] (2023)	SSI	VCs, DLT	ERC721	Not mentioned	Concept description and example	Validation in Real-World Environments
[70] (2023)	GAIA-X, IDSA	VCs, VPs	PKI, TLS	eIDAS 2.0	Use case	Identification and Identity Governance
[71] (2024)	GAIA-X	Wallets, VCs	RBAC	Considered for further research	Requirements identification	Interoperability and Architecture Extension
[72] (2024)	GAIA-X, IDSA, EDC, Pontus-X	Wallets, VCs, VPs	JSON-LD	eIDAS	Use case	Interoperability and Architecture Extension
[73] (2024)	SSI	VCs	OAuth 2.0, Ethereum, ERC735, ERC734, FIWARE keyrock	eIDAS2, GDPR	Prototype implementation	Validation in Real-World Environments
[74] (2024)	GAIA-X, IDSA, EDC	Wallets, VCs, VPs	JSON-LD, TRAIN, DNSSEC	eIDAS	Use case	Validation in Real-World Environments
[75] (2024)	IDSA, IDS connector	VCs, VPs	RFID, JSON-LD, DID	Not mentioned	Architecture presentation	Validation in Real-World Environments
[76] (2024)	GAIA-X	VCs	Smart Contracts, Ethereum, VM	Not mentioned	Prototype implementation	Validation in Real-World Environments
[77] (2024)	Self	VCs	DIDComm, JSON-LD	GDPR	Research prototype	Interoperability and Architecture Extension
[78] (2024)	GAIA-X, EDC	Wallet, VCs, VPs	IOTA, smart contracts	Not mentioned	Use case implementation	Validation in Real-World Environments
[79] (2025)	GAIA-X	VCs, VPs	OIDC, universal DID resolver, TRAIN	eIDAS	Prototype implementation	Interoperability and Architecture Extension
[80] (2025)	Self, FIWARE	VCs, VPs	OIDC, XACML, JSON-LD, keycloack iSHARE	Not mentioned	Prototype implementation	Identification and Identity Governance
[81] (2025)	GAIA-X, IDSA	VCs, VPs, DLT	Hyperledger Aries Fabric, Medium Security Policy Language (MSPL)	Not mentioned	Experimental deployment	Interoperability and Architecture Extension

Q1. *Architecture* Out of the 18 reviewed articles, 11 (≃61%) explicitly build their architectural proposals on top of the Gaia-X framework, often in combination with the Eclipse Dataspace Connector or IDS connector, both precursors of the Eclipse Dataspace Components (EDC), and in one case the Pontus-X connector is also considered. The prevalence of Gaia-X is partly explained by the fact that it was included as a search criterion in the systematic review, which naturally increased its representation. Nevertheless, even there are contributions that do not ground their architectures directly in Gaia-X, they still reference it as a relevant initiative, underscoring its central role as the main ecosystem of reference.

Four contributions (≃22%) combine Gaia-X with IDSA, while one is only based on the later (IDSA) and another one based on FIWARE. The remaining works describe project-specific or self-defined architectures. Naturally, as the DSSC Blueprint was first published in late 2023, none of the works refer to it. Finally, four of the works focus more on SSI architectures than on data space concept.

*Q2. Identity and Credential Governance* In almost all works (≃94%), credential governance relies on the concept of Verifiable Credentials (VCs) and Verifiable Presentations (VPs), usually associated with a digital Wallet. In a few cases, extensions or alternative models appear (e.g., three contributions include DLT in blockchain contexts), but the Verifiable Credentials remains the de facto standard across the literature. Only one of the analysed works uses X.509 certificates. These results suggest that all the works are aligned with SSI paradigm although they do not refer to the ToIP Stack*.*

*Q3. Identity and Credentials Technologies and Standards* More than half of the articles (10/18, ≃56%) use OAuth 2.0 and/or OpenID Connect as their main authorization and federation layers. About one third incorporate blockchain/DLT infrastructures (e.g., Hyperledger, Ethereum, IOTA), mainly for credential traceability or smart contracts. Other technologies include decentralized messaging protocols (e.g., DIDComm, SIOPv2) and policy languages (e.g., MSPL, XACML), showing a heterogeneous landscape.

*Q4. Compliance* The most frequently referenced regulation is eIDAS/eIDAS2, explicitly mentioned in 12 out of 18 articles (≃67%). Among these, four works go further by considering eIDAS2 requirements in advance. GDPR is explicitly addressed in only 4 articles (≃22%), and often in a marginal way. One third of the contributions do not reference any regulatory framework at all, suggesting a persistent gap between technical design and compliance with privacy regulations.

*Q5. Evaluation and Maturity* Half of the works (9/18, 50%) remain at the level of use cases or proof-of-concepts. Five (≃28%) report prototype implementations, while four (≃22%) are limited to conceptual or architectural descriptions. Only three contributions include validation in real-world environments such as manufacturing or eGovernment. This confirms that overall technological maturity is still low and that systematic evaluation metrics (e.g., usability, security) are rarely applied.

*Q6. Implementation Challenges and Open Issues* The reviewed works reveal several open challenges, ranging from the need for pilots and validation in real settings to interoperability limitations and persistent problems in identity governance. Overall, scalability, interoperability, and ecosystem alignment remain unresolved.

• **Validation in Real-World Environments:** highlighted in 7 articles (≃39%), pointing to the lack of pilots, feedback from real deployments, and testing in operational contexts.
• **Interoperability and Architecture Extension:** reported in 8 articles (≃44%), emphasizing the need to integrate multiple credential formats, extend protocols, and adapt architectures to new domains.
• **Identification and Identity Governance:** explicitly discussed in 3 contributions (≃17%), focused on challenges in identity matching (e.g., eIDAS), alternative identification methods, and standardized semantics and trust levels.

In summary, interoperability limitations and the transition from prototypes to operational deployments emerge as the most pressing bottlenecks, followed by the alignment of identity governance models with ecosystem-wide requirements.

***Limitations*** As a result of the significant number of standards and protocols combined with its still evolving state, the terms used across organizations or domains are different and the concepts misaligned. This lack of consensus can limit our results as it hinders the query’s coverage. Because the focus of this work is on Gaia-X–aligned data spaces, we believe that the approach we took of combining targeted search queries with broader terms, and then employing a posteriori screening to prioritize works that address data spaces and include information on credentials, provides a balanced methodology given the current heterogeneity. Another limitation is the exclusion of other identity and trust frameworks, such as the iSHARE Trust Framework. However, we chose to focus exclusively on Gaia-X because of its comprehensive, systemic approach to infrastructure sovereignty, which provides a uniquely integrated model for decentralized and federated governance.

### 5.7 Conclusions and future research directions

The systematic literature review has examined identity and credential management in the context of data spaces aligned with Gaia-X. The analysis shows that the topic is still being addressed at a high conceptual and architectural level in most of the surveyed papers, as usually suggested by the basic credential trust model considered (Q2) and the short list of technologies and standards (Q3). The ambition of Gaia-X and related efforts is evident: to establish a common European ecosystem that guarantees interoperability, sovereignty, and trust. However, the results also reveal that although Gaia-X is increasingly penetrating the academic and practitioner communities (Fig. 8), there is still a long road to walk until Gaia-X compliant data spaces in operation are the subject of academic publications. Several critical gaps can be identified.

First, standards and specifications remain fragmented and evolving. Although bodies such as W3C, DIF, OpenID Foundation, ETSI, ISO/IEC, and the Gaia-X Association itself are producing relevant guidelines, there is no single, mature standard that has achieved broad adoption across data spaces as reflected in the surveyed literature. The coexistence of multiple candidate protocols and models leads to uncertainty and hinders the development of interoperable solutions. Nevertheless, the overview of the landscapes presented in Sections 2, 3, 4 show that convergence and alignment among stakeholders are progressively strengthening.

Second, the literature and initiatives surveyed often focus on theoretical architectures, reference models, and pilot tools, but very few works demonstrate robust evaluation in real-world scenarios. Most contributions stop at proof-of-concept implementations or pilot projects with limited scope, without addressing scalability, usability, or operational deployment in production environments. This lack of empirical validation makes it difficult to assess the actual feasibility of the proposed solutions. On the other hand, this result is not unexpected within the surveyed works, as the Digital Europe Program (DEP) started to operate in late 2020 and the first Gaia-X release (Elbe) was in 2021.

Third, the ecosystem itself is diffuse and complex. Numerous initiatives: Gaia-X, IDSA, FIWARE, BDVA, DSSC, SIMPL, EBSI, among others, are advancing in parallel, sometimes overlapping in scope and outputs. While this diversity reflects strong momentum and interest, it also creates barriers to entry for new stakeholders and risks duplication of efforts. The absence of a clear roadmap that aligns all these efforts under consistent standards and governance models further complicates adoption. Researchers, theoretical or in the context of data spaces development, should try to clearly position their proposals within both the data spaces and SSI ecosystems (e.g., taking as reference the DSSC and ToIP specifications).

Based on the systematic literature review and the current state of data spaces and self-sovereign identity, several promising avenues for future research emerge.

#### 5.7.1 Standardization and interoperability convergence and ecosystems harmonization

*Harmonizing Initiatives.* Research is needed to develop strategies and models for better coordination among the numerous parallel initiatives (Gaia-X, IDSA, FIWARE, BDVA, DSSC, SIMPL, EBSI). This could involve identifying optimal collaboration models, defining clear scopes, and establishing shared roadmaps to minimize duplication of effort and accelerate adoption.

*Bringing together ToIP and data spaces communities*. Further exploration into how the layers of the ToIP Stack interoperate within the Gaia-X framework and other data space architectures is essential. This includes researching how Layer 1 (public DID utilities) and Layer 2 (digital wallets and agents) effectively support Layer 3 (verifiable credential exchange) in diverse operational scenarios.

*Developing Coordinated and Interoperable Standards.* Research is needed to drive the convergence of the fragmented landscape of standards and specifications from bodies such as W3C, DIF, OpenID Foundation, ETSI, ISO/IEC, and Gaia-X itself. This could involve proposing meta-standards or translation layers that bridge existing protocols and data models, thereby reducing uncertainty and fostering widespread adoption.

*Interoperability Testing Frameworks.* There is a need for robust, systematic testing frameworks and methodologies to assess the true interoperability of different SSI technologies and data space implementations. This includes developing standardized compliance and conformance tests that go beyond theoretical compatibility to practical, cross-platform interoperability.

#### 5.7.2 Real-world evaluation and scalability

*Empirical Validation in Production Environments.* Most current work is limited to proof-of-concept or pilot implementations. Future research should focus on rigorous empirical validation of Gaia-X-aligned data spaces and SSI solutions in full-scale, real-world production environments. This includes evaluating performance, scalability, usability, and security under realistic load conditions.

*Longitudinal Studies of Adoption.* Conducting long-term studies on the adoption patterns, benefits, and challenges of SSI and data space technologies in various industrial sectors (e.g., healthcare, energy, manufacturing) can provide crucial insights into their practical impact and areas for improvement.

*Data Product Lifecycle Management.* Further research into the identity and credential implications throughout the entire lifecycle of a "data product" within a data space, from its creation and publication to its exchange, usage, and eventual archival or deletion, ensuring continuous trust and compliance.

## 6 Conclusions

Gaia-X aligned data spaces are still in an early stage of consolidation, but the literature shows their strategic importance for achieving secure, sovereign and interoperable data sharing in Europe. Identity and credential management consistently rely on self-sovereign identity paradigms, with verifiable credentials, wallets and decentralized identifiers emerging as the de facto mechanisms to implement trust in these ecosystems. However, fragmented standards, limited real-world pilots and insufficient alignment with regulatory frameworks such as GDPR and eIDAS 2.0 reveal that technological maturity remains low and large-scale interoperability is not yet achieved. Addressing these gaps through coordinated standardisation, systematic interoperability testing, and evaluation in production environments will be essential for Gaia‑X to support trustworthy data exchange at scale.

The presented ecosystem landscape and the systematic literature review jointly provide a structured foundation for understanding how Gaia‑X, self‑sovereign identity and data spaces intersect in practice. By organizing the state of the art across these three pillars and mapping concrete technologies, roles and governance models, the work turns a highly fragmented body of knowledge into an actionable reference for both researchers and practitioners. The identification of open issues around interoperability, trust assurance, and standard alignment further clarifies where current solutions fall short and where targeted innovation is most needed.

These contributions naturally open several promising research lines, including the design of interoperable identity and credential frameworks across heterogeneous data spaces, the development of verifiable trust and conformity assessment mechanisms, and the evaluation of Gaia‑X‑aligned architectures under real-world regulatory and operational constraints. Advancing these directions will not only help close the gap between conceptual models and deployable infrastructures, but will also strengthen digital sovereignty and trust in cross‑border data sharing, making this a timely and impactful area for future research.

## Ethics Statement

The authors have read and follow the *ethical requirements* for publication in Data in Brief and confirm that the current work does not involve human subjects, animal experiments, or any data collected from social media platforms.

## CRediT Author Statement

**Roi Sánchez Serna, Carla López García, Ana I. González-Tablas**: Conceptualization, Methodology, Investigation, Writing—original draft, Writing—review & editing. All authors contributed equally to this work, and have approved the final version of the manuscript.

## Acknowledgements

The work of C. López García and A.I. González-Tablas has been supported by the project “Energía Inteligente para todos: un enfoque desde los gobiernos locales – EITEL” (TSI-100121-2024-73), funded by the Spanish Ministry for Digital Transformation and Public Service. The work of R. Sánchez Serna and A.I. González-Tablas has been supported by the Spanish National Cybersecurity Institute (INCIBE) grant “APAMciber” within the framework of the Recovery, Transformation and Resilience Plan funds, financed by the European Union (Next Generation). A.I. González-Tablas’ work has also been supported by the grant RED2024-154240-T funded by MICIU/AEI/10.13039/501100011033.

## Declaration of Competing Interest

The authors declare that they have no known competing financial interests or personal relationships that could have appeared to influence the work reported in this paper.

## Footnotes

1 https://knowledge-base.inspire.ec.europa.eu/index_en, last access September 2025.

2 https://dssc.eu/, last access September 2025.

3 https://gaia-x.eu/, last access September 2025.

4 https://data-spaces-business-alliance.eu/, last access September 2025.

5 https://digital-strategy.ec.europa.eu/en/policies/data-spaces, last access September 2025.

6 https://simpl-programme.ec.europa.eu/, last access September 2025.

7 https://interoperable-europe.ec.europa.eu/collection/european-interoperability-reference-architecture-eira/solution/egovera-upscale, last access September 2025.

8 https://interoperable-europe.ec.europa.eu/collection/semic-support-centre/data-spaces, last access September 2025.

9 https://interoperable-europe.ec.europa.eu/collection/semic-support-centre/data-spaces, last access September 2025.

10 https://internationaldataspaces.org/, last access September 2025.

11 https://www.health-x.org/en/home, last access September 2025.

12 https://omega-x.eu/, last access September 2025.

13 https://www.fiware.org/, last access September 2025.

14 https://fiware-orion.readthedocs.io/en/master/, last access September 2025.

15 https://fiware-datamodels.readthedocs.io/en/stable/ngsi-ld_howto/index.html, last access September 2025.

16 https://swagger.lab.fiware.org/?url=https://raw.githubusercontent.com/Fiware/specifications/masterOpenAPI/ngsiv2/ngsiv2-openapi.json, last access September 2025.

17 https://docs.gaia-x.eu/#/framework, last access September 2025.

18 https://gaia-x.eu/services-deliverables/digital-clearing-house/, last access September 2025.

19 https://docs.gaia-x.eu/policy-rules-committee/policy-rules-labelling/22.11/, last access September 2025.

20 https://bdva.eu/, last access September 2025.

21 https://bdva.eu/federation-of-i-spaces/, last access September 2025.

22 https://dataports-project.eu/, last access September 2025.

23 https://www.i3-market.eu/, last access September 2025.

24 https://www.eclipse.org/, last access September 2025.

25 https://github.com/eclipse-edc/Publications, last access September 2025.

26 https://projects.eclipse.org/projects/technology.dataspace-drp, last access September 2025.

27 https://www.tmforum.org/, last access September 2025.

28 https://standards.cencenelec.eu/ords/f?p=205:7, last access December 2025.

29 https://trustoverip.org/, last access September 2025.

30 https://ec.europa.eu/digital-building-blocks/sites/spaces/EUDIGITALIDENTITYWALLET/pages/694487738/EU+Digital+Identity+Wallet+Home, last access December 2025.

31 https://ec.europa.eu/digital-building-blocks/sites/spaces/EUDIGITALIDENTITYWALLET/pages/694487808/What+are+the+Large+Scale+Pilot+Projects, last access December 2025.

32 https://ec.europa.eu/digital-building-blocks/sites/spaces/EBSI/pages/447687044/Home, last access September 2025.

33 https://w3.org, last access September 2025.

34 https://identity.foundation/, last access September 2025.

35 Draft: https://identity.foundation/didcomm-messaging/spec/v2.01, last access September 2025.

36 Draft: https://identity.foundation/credential-manifest/v1.0.0, last access September 2025.

37 https://didcomm.org/issue-credential/3.0/, last access September 2025.

38 https://didcomm.org/present-proof/3.0/, last access September 2025.

39 https://openid.net/foundation/, last access September 2025.

40 https://openid.net/developers/how-connect-works/, last access September 2025.

41 https://www.lfdecentralizedtrust.org/, last access September 2025.

42 https://www.lfdecentralizedtrust.org/projects/fabric, last access September 2025.

43 https://www.lfdecentralizedtrust.org/projects/hyperledger-indy, last access September 2025.

44 https://lf-hyperledger.atlassian.net/wiki/spaces/ANONCREDS/overview, last access September 2025.

45 https://framework.ishare.eu/, last access March 2026..

46 https://eudi.dev/2.5.0/architecture-and-reference-framework-main/, last access December 2025.

47 https://www.dataspaces-radar.org/radar/, last access December 2025.
